# Intravenous Lipid Emulsion Rescue Therapy in a Child with Carisoprodol Overdose

**DOI:** 10.7759/cureus.6250

**Published:** 2019-11-27

**Authors:** Madhuradhar Chegondi, Aditya Badheka, Swathi Chacham, Madelyn Kahana

**Affiliations:** 1 Pediatrics, University of Iowa Stead Family Children's Hospital, Iowa City, USA; 2 Pediatrics, University of Iowa Hospitals and Clinics, Iowa City, USA; 3 Pediatrics, All India Institute of Medical Sciences, Rishikesh, IND; 4 Pediatric Critical Care Medicine & Anesthesiology, Nemours Children's Hospital, Orlando, USA

**Keywords:** child, carisoprodol, intravenous lipid emulsion

## Abstract

The use of intravenous lipid emulsion (ILE) therapy in children with carisoprodol toxicity was not described previously. We report the case of an adolescent female who presented to our pediatric intensive care unit with unresponsiveness and respiratory depression. The patient recovered immediately following ILE therapy and subsequently admitted having carisoprodol overdose.

## Introduction

Intravenous lipid emulsion (ILE) therapy is widely used in local anesthetics associated systemic toxicity (LAST) [1]. ILE has been suggested as a potential treatment for other lipid-soluble drugs as well [1,2]. The hypothesis is the ILE may partition the lipid-soluble drug into lipid that may significantly alter its pharmacokinetics [1]. We describe successful ILE therapy in an adolescent female with carisoprodol drug overdose.

## Case presentation

A 15-year-old previously healthy female was admitted to our tertiary care pediatric intensive care unit (PICU) from a community emergency room with unresponsiveness. She was found by her parents in an unresponsive and tremulous state on the morning of admission. Emergency rescue was called and upon their arrival, the patient was intubated at the scene for significant respiratory depression. The patient was then transported to a local emergency department, and en route she was given one dose 1 mg lorazepam (0.02 mg/kg) for tremulousness, suspected to be seizure-related, with no response. Upon arrival to the outside emergency room, she was unresponsive, normothermic, tachycardic with a heart rate of 150 bpm and with mild hypertension and the blood pressure of 130/84 mmHg. She had symmetric and reactive pupils on examination. She received an additional 1 mg of lorazepam for ongoing tremulous movements with no response. She had an unremarkable brain computerized tomography scan and cerebrospinal fluid studies. She had normal electrolytes and liver function test. In addition, urine toxicology screen, blood alcohol, acetaminophen, and aspirin level were negative. She received a normal saline bolus, and a dose of ceftriaxone was then transferred to our PICU with a diagnosis of status epilepticus and possible meningitis. Just prior to transport, she was started on propofol infusion at 20 mcg/kg/min. At admission to the PICU, her physical examination was notable for normothermia, normotension, and tremulous motor activity. She had no response to painful stimuli but had a normal pupillary examination. Her propofol infusion was discontinued immediately and on follow-up assessment, she was able to localize to pain. Her repeat electrolytes, blood gas, chest X-ray, and electroencephalogram were normal, and electrocardiogram revealed sinus tachycardia with normal PR or QTc intervals. Over the next 10 hours, her neurological status remained unchanged. She was continued on invasive mechanical ventilation and intravenous fluids. Despite her negative toxicology screen, and her family’s denial of any history drug use in the past or significant potential sources of mood-altering drugs in the house, we considered a trial of ILE therapy secondary to the possibility of a drug overdose. She was started on intravenous 20% lipid emulsion at a dose of 1 mL/kg over one hour. By the end of lipid infusion, the patient had such a dramatic improvement in her sensorium, she was extubated, and became quite verbal admitting that she ingested about forty 350 mg of each tablet of carisoprodol (14 gm), which was used by her father for chronic lower back pain.

## Discussion

There is limited data regarding the use of ILE as rescue therapy [1]. Two decades ago, Weinberg showed the efficacy of ILE therapy in animal models during resuscitation following local anesthetic toxicity [2]. The first clinical application of ILE therapy reported in an adult patient with presumed bupivacaine-induced cardiac arrest who initially failed with standard resuscitative measures and completely recovered shortly after lipid emulsion infusion [2,3]. Its usage is mainly limited to anesthesiology, and there are few case reports in adult patients suggesting successful recovery of local anesthetic systemic toxicity following ILE therapy administration [3,4]. Ludot et al. [5] reported successful ILE rescue therapy in a child with local anesthetic-induced ventricular arrhythmia following posterior lumbar plexus block. Spence [6] reported a reversal of neurological symptoms in an obstetric patient, with a rapid administration of ILE. More recently, ILE therapy also used successfully in patients presenting with non-local anesthetic medication toxicity such as beta-blockers, calcium channel blockers, and tricyclic antidepressants [2,7].

ILE is a mixture of medium and long-chain fatty acid triglycerides. Even though its exact mechanism is unclear, the proposed mechanisms of the lipid sink phenomenon and the dynamic shuttle effect explain its clinical effects [8,9]. According to the lipid sink phenomenon, following administration of ILE, by creating a concentration gradient, all lipophilic substances are drawn from target tissues into the expanded lipid medium and in theory, toxicity is reversed [8]. However, the dynamic shuttle or scavenging effect of ILE depends on the lipid’s drug-binding properties and its redistributive effects [9]. In patients with other forms of drug toxicity with unsuccessful resuscitative efforts, ILE therapy may be reasonable therapy [10]. The American College of Medical Toxicology [11] suggested that the decision to use ILE in the management of a highly lipid-soluble xenobiotic in conjunction with supportive therapies in a hemodynamically unstable patient is dependent on the bedside clinician.

Carisoprodol is a centrally acting muscle relaxant and sedative with the potential for abuse [12]. It is commonly used as adjunctive therapy for acute painful muscle spasms associated with musculoskeletal conditions [12]. Its exact mechanism of action is unknown. It is metabolized in the liver and produces an active metabolite meprobamate. It has an elimination half-life of eight hours and excreted mainly via the renal route. Both carisoprodol and its major metabolite, meprobamate, produce benzodiazepine-like effects by interacting with gamma-aminobutyric acid (GABA) receptors [12]. When large doses of carisoprodol are ingested, central nervous system depression and respiratory failure occur, which were predominant features of our case. Anticholinergic toxidrome features, prolonged seizures, and coma were also reported in an adult patient with large dose ingestion [13]. There is no specific antidote for carisoprodol toxicity. Supportive care in the intensive care setting is the mainstay of therapy, including maintaining an airway, mechanical ventilation, and intravenous hydration to ensure adequate drug elimination. If a patient presents immediately after ingestion, gastric lavage and activated charcoal can be given. Other measures include flumazenil, and hemoperfusion [13,14]. In our extensive literature search, we could not find the use of ILE therapy in carisoprodol toxicity previously. Our patient admitted with respiratory depression and coma, and we were unable to identify the specific drug ingestion. Given no change in clinical condition over 10 hours, we elected to treat our patient with 1 mL/kg ILE. Current literature supports giving larger doses of intravenous lipid for drug ingestion rescue than was used in this child. Common starting doses reported are 1-2 mL/kg as a bolus over three to five minutes followed by 0.25 mL/kg/hr as an infusion continued until 10 minutes after the stable hemodynamics of the patient [15]. It is also suggested that one can repeat the bolus dose and increase the infusion to 0.5 mL/kg/hr if the patient remains hemodynamically unstable. The maximum amount of lipid recommended as an initial rescue dose that we could find in the literature is 12 mL/kg over 30 minutes [15]. There were no reported adverse effects of ILE in the doses recommended by the American Society of Regional Anesthesia and Pain Medicine (ASRA) [16]. However, the safety profile of ILE is unclear with higher doses other than the recommended as per the ASRA guidelines [16]. Carisoprodol is a lipophilic drug. We believe that due to its lipophilic nature, the lipid emulsion acted as a lipid sink for carisoprodol and reversed the altered clinical condition of the patient. Unfortunately, blood levels of carisoprodol were not available to us for clear objective evidence of this child’s response to lipid therapy.

## Conclusions

ILE therapy is a life-saving measure for LAST. Since it has no significant side effects with the recommended doses and readily available to use, we conclude that ILE therapy can be considered in carisoprodol toxicity and other conditions when drug ingestion is strong clinical suspicion. There is a need for more experience using ILE usage in drug toxicities other than local anesthetics. We hope that this report brings this safe intervention additional attention in its potential uses outside LAST.
